# From mRNA stabilisation to mirror life: Biological orthogonality, incremental risk, and anticipatory governance

**DOI:** 10.3389/fbioe.2026.1833212

**Published:** 2026-09-03

**Authors:** Christopher H. Lean, Russel M. Vincent, Kate E. Lynch, Euzebiusz Jamrozik, Paul R. Jaschke

**Affiliations:** 1 Philosophy, Macquarie University, Sydney, NSW, Australia; 2 Australian Research Council Centre of Excellence in Synthetic Biology, Macquarie University, Sydney, NSW, Australia; 3 Macquarie Medical School, Macquarie University, Sydney, NSW, Australia, Sydney, NSW, Australia; 4 School of History and Philosophy of Science, The University of Sydney, Sydney, NSW, Australia; 5 Ethox and Pandemic Sciences Institute, University of Oxford, Oxford, United Kingdom; 6 Department of Infectious Diseases at The Peter Doherty Institute & Royal Melbourne Hospital Department of Medicine, University of Melbourne, Melbourne, VIC, Australia; 7 Monash Bioethics Centre, Monash University, Melbourne, VIC, Australia; 8 Macquarie University School of Natural Sciences, North Ryde, NSW, Australia

**Keywords:** biological orthogonality, biosecurity, mirror life, mRNA, stabilization

## Abstract

Molecular stabilisation of mRNA is central to the development of therapeutics. Stabilisation is gained through modifications that cause mRNA to resist degradation, evade immune recognition, and prolong expression. These modifications introduce degrees of biological orthogonality, which, in this context, is the extent to which a molecule becomes invisible to the natural systems that would otherwise process or eliminate it. We propose that orthogonality provides a framework for understanding biosecurity risks across the spectrum of synthetic nucleic acid modification, from current mRNA therapeutics to mirror life. We develop this framework through a five-stage continuum from engineered mRNA to fully orthogonal mirror-life systems, using the 2024 scientific moratorium that was recommended for mirror life as a biosecurity governance endpoint. Across this spectrum, there is an increasing degree to which engineered genetic material can resist degradation and evade immune recognition, and a threshold at which this genetic material could be replicated. mRNA has minimal biosecurity issues because it is detectable, immunologically decomposable and unable to replicate, whereas mirror life would be highly undetectable, minimally decomposable, and replicable. Orthogonality, however, does not appear only at these extremes, and we suggest that biosecurity-relevant risks can accumulate progressively as we engineer greater orthogonality. Orthogonality should be a significant factor in designing biosecurity governance, alongside sequence screening and product-level assessments. We suggest a trajectory-focused oversight that monitors, across research programmes, the properties that turn orthogonality into risk and triggers staged reviews as they advance. The properties in question are persistence, reflected in half-life extension, nuclease resistance, and environmental persistence, along with invisibility to recognition and surveillance and the capacity for autonomous replication.

## 1 Introduction

Messenger RNA (mRNA) is a single-stranded ribonucleic acid that carries protein-coding instructions from DNA to the ribosome. Synthetic mRNA exploits this natural mechanism to deliver transient protein-coding instructions directly into cells without genomic integration, allowing for protein expression without germline changes. The success of the mRNA vaccines during the COVID-19 pandemic demonstrated that synthetic mRNA can be deployed at a global scale, opening pathways for new applications in human health. These include vaccines targeting further infectious diseases (e.g., RSV) and cancer (e.g., melanoma), to therapeutics such as growth factors to treat heart disease and protein replacement for genetic conditions such as cystic fibrosis (Wilson et al., 2023; Bidram et al., 2021; Qin et al., 2022). Each of these applications must contend with a common biological obstacle: living systems have evolved highly effective mechanisms for detecting and degrading exogenous RNA. For mRNA specifically, this bottleneck is acute. It is attacked by ribonucleases throughout the body and degraded by innate immune mechanisms. These biological mechanisms are highly attuned to remove RNA as foreign RNA is associated with viruses and infections. Life has evolved a diverse range of mechanisms for degrading mRNA. mRNA triggers a range of immunological responses, including TLR3, TLR7, and TLR8 and the RIG-I and MDA5 pathways. RNases are abundant throughout the body, in tissues, and in the intracellular and extracellular environments. These mechanisms are often deeply embedded across taxa, with viruses evolving mechanisms to package mRNA that evade immune responses and organisms possessing mechanisms to destroy these packages. This is a problem because for mRNA therapeutics to be effective, they must remain in the cell long enough to be translated.

The effective delivery of mRNA therapeutics thus requires significant alterations to natural mRNA sequence, architecture, and cellular location to overcome this problem. Two broad strategies have been developed to address this: lipid nanoparticle (LNP) encapsulation, which creates a physical barrier between the mRNA and the extracellular degradative environment and provides a route–through endocytosis–to enter the cytoplasm and access the protein-synthesis machinery; and molecular stabilisation, which involves modifying the mRNA itself to evade immune detection and enzymatic degradation. We are focused on molecular stabilisation rather than delivery systems such as LNPs, as we are examining the engineering of novel genetic material rather than shielding genetic material with a physical barrier. Methods for molecular stabilisation include N1-methylpseudouridine modification, cap-1 structures, codon optimisation, and human α-globin 5′UTR and 3′UTR changes (Szabó et al., 2022). Base modification strategies (such as N1-methylpseudouridine modification) draw on viral biology: Karikó et al. (2005) observed that viral genomes carry high concentrations of base modifications that reduce innate immune activation. Current therapeutic UTR designs, by contrast, are derived from human sequences or engineered synthetic constructs, not viral architectures (Metkar et al., 2024).

Recent advances in synthetic nucleic acid technology have lowered the barriers to creating increasingly persistent and immunoevasive RNA molecules. Improving mRNA persistence increases the efficacy of mRNA interventions. The natural half-life of mRNA is regulated in the cell by binding proteins, regulatory RNAs (miRNA and lncRNA) and alternative polyadenylation in the 3′UTR of genes. This natural transience is one of mRNA’s most important safety features because mRNA is short-lived and its expression window is limited, reducing the risk of prolonged immunological stress, off-target protein expression, and genomic integration. A highly persistent mRNA architecture would depart from this property and raise new ethical issues, potential ecological risks, and arguably require significant public engagement prior to widespread use.

This creates a trade-off for mRNA stabilisation. The same modifications that increase therapeutic efficacy by extending the window of protein expression, reducing immunogenicity, and improving resistance to degradation are precisely those that introduce novel biological behaviours whose long-term consequences are poorly understood. This is the primary issue with designing for orthogonality. Orthogonality involves creating a system part with a specific function that can be altered and optimised with little effect on the rest of the system (Woolfson, 2026). The problem is that detection, decomposition, or compensatory changes are significant roles that biological systems need to perform when incorporating novel molecules. So, optimising for persistence is not a straightforward improvement; it involves accepting increasing degrees of biological orthogonality that accumulate incrementally and which may not trigger regulatory concern until a risk threshold has already been crossed.

This paper develops an analogy to the recent scientific calls for a moratorium on “mirror life”: synthetic biological systems constructed from nucleic acids and proteins of opposite chirality, which are believed to be completely orthogonal to all living organisms. Current mRNA stabilisation strategies largely exploit mechanisms already found in nature, including archaeal base modifications and human UTR sequences. These typically work within and are processed by standard cellular systems, making them magnitudes less risky than mirror life. They do, however, represent two ends of the orthogonality spectrum currently discussed in novel biotic design. Both involve engineering genetic material to resist degradation, evade immunological recognition, and persist in biological environments in ways that biology has not previously encountered. Mirror life, therefore, provides a useful “end case” for how mRNA stability should be ethically designed and governed.

### 1.1 Mirror-life and precaution

All life on Earth is homochiral: nucleic acids are built from right-handed sugars (D-form), and amino acids are left-handed (L-form). Mirror-life entails the creation of not just the chiral form of drugs or proteins, but an entire mirror transcription system (mirror-handed genetic codes and polymerases to read them), and ultimately a mirrored organism including DNA replication, protein translation, lipid synthesis, and cofactor synthesis. Upon its initial proposal and discussion, there was an incredible amount of enthusiasm for such a creation, as seen in Ed Regis and George Church’s book *Regenesis* (Church and Regis, 2014). They proposed that mirror life could be a biocontained system that would not interact with or affect other life, functioning as a sandbox for experimentation (Schmidt, 2010). It could allow the creation of a largely separate industrial biology, in which industrial organic systems would not interact with the rest of life or be susceptible to infection or degradation. It would allow exploration of biological possibilities, helping us better understand why life has the chirality it does and the origins of the genetic code.

The enthusiasm for mirror life has been tempered by the release of a technical report and its companion piece in *Science*, which argued that mirror life poses unique biosecurity threats (Adamala et al., 2024). What was missed in the early discussion of mirror life was that an autonomous, non-degrading replicating biotic system is highly risky. Creating mirror life forms could lead to their widespread proliferation and persistence in the environment, as they are likely to evade natural predation and immune responses due to their reversed chirality, leading them to avoid existing microbial control agents, such as phages and antibiotics, making containment and mitigation extremely challenging. This could lead to mirror-life species that could cause widespread, potentially lethal infections in humans, animals, and plants, resulting in ecological and health-related harms.

These fears have led to concrete recommendations. Adamala et al. (2024) explicitly call for a prohibition on research aimed at creating mirror bacteria, and for funders to make clear they will not support such work. Beyond this research prohibition, they advocate for developing governance frameworks to regulate enabling technologies, including monitoring systems for mirror oligonucleotides and precursor materials, and regulations to prevent the production of mirror genomes and proteomes. They further recommend initiating international collaborative discussions involving scientists, policymakers, regulators, industry, and civil society to establish effective oversight, and that these measures be regularly reviewed as science advances and new pathways to mirror life are identified. The nature of these recommendations goes beyond standard precautionary governance: what is proposed is anticipatory research restraint, targeting specific enabling trajectories before the capability is realised, to prevent the creation and dissemination of the relevant technologies.

mRNA stabilisation lacks these extreme risks as it does not replicate, cannot evolve, and lacks the biological autonomy of mirror life. Nonetheless, the mirror-life indicates potential risks of persistent, immune-evading genetic material. While the analogy is not about the equivalence of danger, it reminds us that biological stability is not neutral, as biological systems evolved to degrade native and foreign RNA, and the circumvention of these mechanisms should be approached with caution. Natural interactions between cells and mRNA define mRNA’s half-life, which ultimately can influence many cellular homeostatic interactions downstream. Circumvention of degradation pathways, therefore, could risk disrupting regulatory dynamics beyond immune function. Stabilisation strategies represent one region of a broader spectrum of synthetic nucleic acid chemistries; understanding where mRNA stabilisation sits within that spectrum, and what lies further along it, is necessary for calibrating the governance response. The rapid evolution of nucleic acid chemistry, spurred by advances in the science and falling costs of synthesising new biotic materials, could outpace governance infrastructure designed to ensure biosecurity and biocontainment.

## 2 Risks in mRNA stabilisation

### 2.1 Immunological and biophysical risks

Given the tight relationship between foreign RNA (including RNA we may want to introduce) and viruses, it is unsurprising that mRNA triggers significant immune responses. Foreign RNA is inflammatory. Toll-like receptors (TLR3, TLR7, TLR8), RIG-I, and MDA5 detect pathogen-associated RNA structures and motifs. Natural mRNA is rapidly degraded by RNases both extracellularly and intracellularly. Each endogenous mRNA has a characteristic half-life that is regulated by cellular needs. Synthetic modifications successfully reduce this degradation by reducing recognition and blocking degradation mechanisms. The persistence of mRNA beyond the point of expression due to immune invasion could, however, pose immunological and biophysical risks that warrant exploration.

Crucial questions for the development of mRNA therapeutics concern what immune responses novel architectures can be expected to trigger. To determine whether novel mRNA therapeutics will trigger a significant immune response, it will be crucial to identify whether responses are triggered by the cargo sequence, the stabilisation modifications, or the delivery system. Current evidence indicates that all these components, to differing degrees, can trigger an immune response. Lipid nanoparticles have been recognised as causing innate immune responses and mechanistic evidence shows that ionizable lipid nanoparticles can induce responses from toll-like receptor 4, even when there is no mRNA within the nanoparticle (Lee et al., 2023; Zelkoski et al., 2025). Evidence that nucleoside-modified mRNA itself induces immune responses remains limited, though it cannot be ruled out. While nucleoside modifications, such as pseudouridine, are designed to reduce immune responses, they do not prevent immune sensing (Xie et al., 2023). Additionally, in mRNA vaccines, the coding region for the viral antigen is designed to induce an immune response. Depending on which of these mechanisms induces an immune response, the risks involved with repeat exposures to differing novel mRNA-based therapeutics will differ.

Increased persistence also raises the possibility of the accumulation of modified mRNA fragments in tissues. Evidence from mRNA COVID-19 vaccines indicates that vaccine components can persist in the body for at least 30 days, having been identified in lymph nodes following vaccination and in cardiac tissue in cases of post-vaccination myocarditis (Krauson et al., 2023). While natural mRNA rapidly degrades, a much more stable system might yield prolonged protein expression, potentially moving mRNA therapeutics closer to gene-therapy risk profiles. Such novel architectures will require significant testing to ensure they do not cause significant injury. Who will be the first to be exposed to such architectures? Healthy volunteers, rare-disease patients, children? Ethical considerations around first-in-class trials become more acute as stabilisation progresses. Beyond initial testing, risks may compound with cumulative exposure and/or environmental contamination with persistent mRNA.

### 2.2 Persistence risks

Although mRNA does not replicate, the biological behaviour of exogenous mRNA introduced into cells must still be understood within the broader evolutionary ecology of RNA systems. Viruses and their hosts are locked in an evolutionary arms race: viruses evolve mRNA sequences and structures that enhance replication, while cells evolve countermeasures to suppress it. A key insight from Nobel laureates Karikó and Weissman was that specific base modifications in mRNA prevented innate immune activation by capitalising on the body’s ability to distinguish self from non-self RNA. Incorporating such modifications into synthetic mRNA causes cells to treat exogenous transcripts more like self RNA, reducing inflammatory responses (Karikó et al., 2005). Their experimental approach was informed by the observation that RNA base-modification type and frequency vary across species, and that some viral genomes carry high concentrations of such modifications (Karikó et al., 2005).

As stabilisation strategies become more elaborate—through modified nucleosides, alternative backbones, and advanced cap analogues—and as effective half-lives lengthen, we should assess risks that scale with persistence. Capture of host genetic material into viral genomes has been documented as a major driver of virus evolution across deep evolutionary timescales (Koonin et al., 2022), and specific cases of host RNA fragment incorporation have been identified in contemporary circulating viruses, including human ribosomal RNA sequences in circulating SARS-CoV-2 genomes (Yang et al., 2022). demonstrating that such events are possible. For any given mRNA therapeutic molecule, which represents a transient addition to the vast pool of cellular mRNAs, such a transfer event is exceedingly unlikely; nevertheless, the probability scales with mRNA longevity.

Whether any captured therapeutic sequence persists in a viral population depends on fitness effects. Insertions that enhance replication, transmission, or immune evasion may be retained; neutral or deleterious segments are rapidly purged by competition. Because viral genomes already explore immense sequence space through mutation and recombination—diversity within a single viral family can rival or exceed that across Bacteria and Archaea (Mihara et al., 2016)—the introduction of additional sequence motifs from therapeutic mRNA is unlikely to expand the landscape beyond what viruses already routinely sample from host RNAs. Indeed, evidence for frequent host to virus sequence transfer is accumulating, including reports of human 18S and 28S rRNA-derived fragments in circulating SARS CoV 2 variants (Yang et al., 2022; Koonin et al., 2022). Given that 18S and 28S rRNAs comprise ∼60–80% of total cellular RNA, copy number is likely a dominant driver of these rare events. From this perspective, mRNA therapeutics would not necessarily introduce more sequence diversity than is already provided by abundant cellular RNAs. What remains understudied is the frequency of such transfers under physiological conditions, and whether specific sequence or structural motifs modulate their likelihood.

It is also important to consider the likelihood that novel RNA base modifications could transfer from therapeutic to human or viral genomes. The predominant base modification in current mRNA therapeutics is N1-methylpseudouridine (m1ψ) (Metkar et al., 2024). This modification does not occur stably outside Archaea, some of which possess the enzymes to catalyse its production. Any hypothetical transfer of a therapeutic sequence to human or viral genomes would transmit only the unmodified base sequence through the copying process, not the altered nucleoside. The modification itself would not persist in the absence of the requisite cellular enzymes acting at that site. Even where a therapeutic employs a modification that also exists in human cells, modification deposition is structure-dependent rather than sequence-dependent alone: PUS1 targets are defined by a local structural motif rather than sequence context in isolation, so a new genomic context would not guarantee modification at the same site (Carlile et al., 2019).

Unlike these base modifications, UTR sequences are encoded directly in the nucleotide sequence and would remain structurally present if captured. This makes sequence-encoded stabilisation a categorically distinct risk subset within Stage 1, even if the probability of any given transfer event conferring advantage remains low. Viral sequences are frequently multifunctional, so any engineered stabilising element (e.g., UTR structures) transferred from a therapeutic mRNA would immediately face selection for compatibility with viral replication, translation control, and packaging. Empirically, 3′ UTRs from divergent picornaviruses can substitute for one another and still support efficient genome replication, despite lacking sequence or secondary-structure homology (Rohll et al., 1995). By analogy, a transferred human-designed UTR would be unlikely to persist unless it conferred a clear advantage within the viral life cycle and would be pruned or reshaped by selection for multifunctionality and genome compactness.

Concern about the excretion of mRNA therapeutics into the environment is frequently raised, yet the improbability of lipid nanoparticles and their mRNA cargo surviving long enough to be excreted intact means there is a notable gap in the literature. Stabilised mRNA engineered to resist environmental RNases could, in principle, persist longer in soils, waterways, or animal tissues, but whether this would constitute a meaningful risk is far from clear. Establishing a baseline for the environmental half-life of non-stabilised mRNA would help calibrate this risk. The closest analogue is the literature on dsRNA-based pesticides, which indicates that environmental dsRNA is generally unstable (hours to days), though durability can increase with protective nanoparticles (Luo et al., 2024). Overall, the current risk appears extremely low; however, low risk is not zero risk, and this distinction remains important for precautionary governance.

### 2.3 Communication risks

A major public communication issue that RNA therapeutics have faced is the public’s fear that RNA vaccines will ‘change their DNA’. A study of COVID vaccine rumours found 22% of respondents had heard the rumour that mRNA vaccines changed people’s DNA (Islam et al., 2021). Another public poll found “nearly half of the public (45%) report having heard the false claim that mRNA vaccines can alter a person’s DNA” (Montero et al., 2025). These are understandable fears. RNA is a ‘genetic material’ like DNA, and significant levels of biological knowledge are needed to understand the differences between RNA and DNA. Language used in the COVID rollouts is also likely to have facilitated the interpretation that RNA is analogous to DNA, with discussion of RNA vaccines as ‘gene therapy’, ‘rewriting DNA’, or ‘gene editing’. mRNA technologies, therefore, face steep communication barriers and may invite threatening interpretations.

The rapid degradation of mRNA has been a tool for reassurance: it allows scientists and regulators to emphasise that therapeutics are transient, non-integrative, and quickly removed from the body. Any attempt to increase persistence must therefore consider its implications for public engagement. If stabilised mRNA lingers in tissues for extended periods, even to achieve greater therapeutic benefit, this may be interpreted as “genetic modification,” regardless of biological accuracy. The more mRNA resembles a long-term genetic intervention, the greater the public suspicion and resistance may be, potentially threatening uptake of future therapeutics. Persistence scales with orthogonality: as molecules become increasingly invisible to degradation and immune recognition systems, their duration of expression extends. This raises the prospect of tissue accumulation and prolonged protein production. In effect, if not in mechanism, such accumulation may approximate the kind of lasting biological change the public associates with permanent genetic modification.

If mRNA therapeutics are interpreted as making permanent changes, this could be perceived as potentially threatening to an individuals’ identity and sense of self. This is due to an importation of genetic essentialist beliefs–the idea that DNA defines an individual’s essential character (Dar-Nimrod and Heine, 2011). Genetic essentialism also entails that genes are deterministic traits and behaviours, an idea so pervasive that it influences perceptions of genetics in the general public (Gould et al., 2012), the law (McSwiggan et al., 2017), medical practices (Lane et al., 2023), behaviour genetic research (Lynch et al., 2019).

Given the pervasiveness of genetic essentialism in the general and professional population, any perception that mRNA therapeutics can permanently impact the genome is likely to invoke essentialist beliefs. The thought is that if someone thinks that their DNA is being altered by using mRNA therapeutics, then they are likely to believe that their underlying sense of self and identity has been altered. This may be negative in some contexts, though it does not necessarily predict resistance to mRNA therapeutics. Patients whose sense of self is organised around a genetic illness (e.g., cystic fibrosis) may embrace successful protein replacement via mRNA as identity-transforming in a positive way. This suggests the communicative challenge about RNA essentialism is not uniform. Essentialist fears about genetic alteration may deter some patients from adopting stabilised mRNA therapeutics, while other patient communities may welcome the identity disruption that general audiences fear. Effective communication strategies will need to be audience-sensitive, recognising that the same therapeutic intervention can carry opposite psychological valence depending on how genetic identity is already implicated in a person’s self-conception.

There is some evidence that media messaging, including the use of metaphors like “blueprint”, “master molecule” and “code” contribute to genetic essentialist beliefs (Nelkin and Lindee, 1995; Nelkin, 2001; Reimann et al., 2025). These metaphors particularly emphasise immutability and determinism. This refers not to how stable DNA remains in our cells over a lifetime, but that the phenotypic *effects* of genes are thought to be determined and inevitable. That is, particular gene variants or combinations of genes are thought to inevitably give rise to certain traits and behaviour, no matter the context. This has led people to believe that ‘genes are our destiny’: that genetic effects are permanent and fixed, a notion that persists in the public imagination, despite decades of criticism from scientists (Dobzhansky, 1959). E.g., people who believe they have “genes for obesity” tend to believe that they will always be obese, and that there’s nothing they can do about it (Ahn and Lebowitz, 2018). Genetic determinism downplays the role of the environment in gene expression, which is almost always interactive and complex. If similar determinist beliefs permeate the public consciousness about mRNA, we may find similar beliefs arise about the inevitability of mRNAs effects, and misunderstandings of the complex role the environment plays in expression. This is a particularly pertinent risk given the narratives of stability, immutability, and permanence surrounding mRNA technology–which could be misinterpreted as implying the immutability of downstream effects of mRNA, rather than the stability of mRNA itself persisting in the body.

Thus, stabilisation poses a dual public communication challenge:

1. Avoiding narratives of permanence and determinism, especially in relation to altering the genome, and
2. Maintaining the public trust that enabled the success of mRNA vaccines, including by acknowledging legitimate public concerns that exist (e.g., myocarditis, stringent mandated policies).

Communication strategies will need to be built into stabilisation research from the outset, including clearer metaphors, visual explanations of degradation dynamics, clear communication about the relationship between DNA and mRNA, and transparency around persistence testing.

## 3 Degrees of orthogonality: synthetic genomes to xenobiology

We have proposed that the risks and ethical issues identified in the creation of mirror life raise much weaker but analogous issues for efforts to stabilise mRNA. This is because these two different applications of synthetic biology sit at two extremes of efforts to create orthogonal biology. mRNA therapeutics pursue selective orthogonality: they must reduce recognition by degradation and immune surveillance systems while remaining fully compatible with translation machinery. Mirror life, by contrast, pursues complete orthogonality and therefore requires its own mirror ribosomes to function. In this section, we highlight how these degrees of orthogonality are being instantiated in current research and provide a preliminary taxonomy of the novel architectures. This is not to say that these are the only ways we could feasibly create orthogonality in biology, or that there is a single linear monotonic measure of orthogonality. But recognising that efforts to increase orthogonality are part of a continuum of engineering is significant for reasoning about communication and material risks at a stage before these technologies are implemented, when envisioning governance can be effective.

Before mapping this spectrum, it is worth delineating two ideas. The first is orthogonality itself, which is a matter of degree because it describes how a molecule escapes the natural systems that would otherwise recognise, process, or eliminate it. Alternatively, the second is biosecurity risk, which depends on three further properties a molecule may acquire as it moves along the spectrum: how far it resists degradation and clearance (persistence), how completely it escapes both biological and technical detection (invisibility to recognition and surveillance), and whether it can replicate autonomously. A highly orthogonal molecule that is rapidly cleared, remains detectable, and cannot self-replicate poses little risk, whereas the same orthogonality becomes hazardous once it is combined with persistence and autonomous replication, as it would be in a self-replicating mirror organism. For this reason, position on the continuum is not by itself a measure of risk. Instead, we treat orthogonality as a dimension of biosafety governance, along which oversight should increase as specific parameters cross risk thresholds, rather than as a simple scale of danger.

### 3.1 Beyond modifications–the synthetic nucleic acid spectrum

**FIGURE 1:**
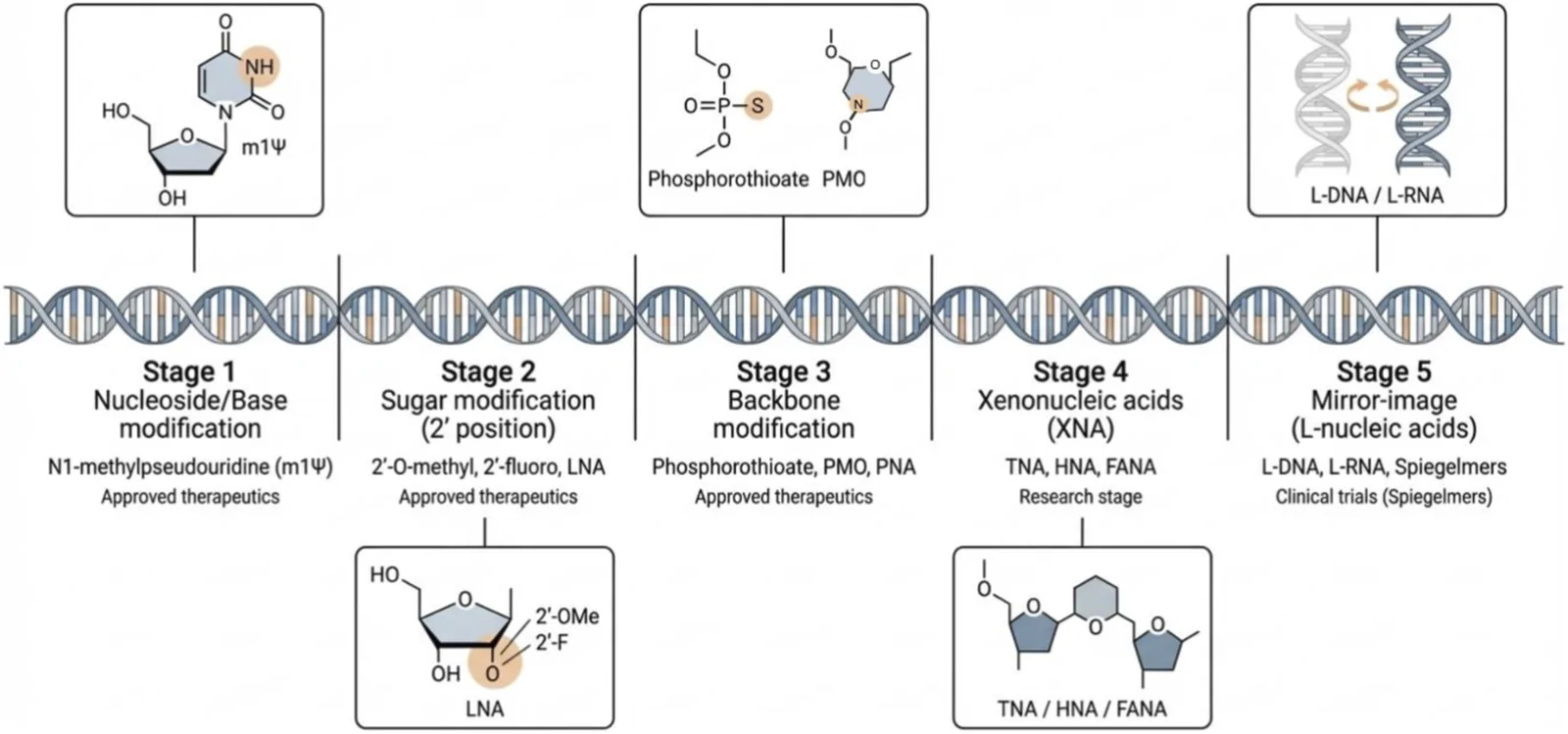
The synthetic nucleic acid modification spectrum: a five-stage framework from natural to fully orthogonal genetic systems. Schematic illustration of the progressive divergence from natural nucleic acid chemistry across five stages of modification. Stage 1: Nucleoside substitutions (e.g., N1-methylpseudouridine) modify the nucleobase while preserving the native sugar-phosphate backbone. Stage 2: Ribose sugar modifications (e.g., 2′-O-methyl, 2′-fluoro, locked nucleic acids) alter the sugar ring to enhance nuclease resistance and binding affinity. Stage 3: Backbone modifications replace the phosphodiester linkage with alternative chemistries such as phosphorothioate, phosphorodiamidate morpholino (PMO), or peptide nucleic acid (PNA) linkages. Stage 4: Xenonucleic acids (XNAs) employ entirely non-natural sugar-phosphate backbones (e.g., threose nucleic acid, hexitol nucleic acid) that require engineered polymerases for replication. Stage 5: Mirror-image (L-configured) nucleic acids possess opposite chirality to natural D-nucleic acids, rendering them invisible to natural enzymes. Approved therapeutics currently span Stages 1–3; Stage 5 Spiegelmers have reached Phase 2a clinical trials.

The stabilisation strategies already introduced represent one region of a broader spectrum of synthetic nucleic acid chemistries; understanding where mRNA stabilisation sits within that spectrum, and what lies further along it, is necessary for calibrating the governance response. The rapid evolution of nucleic acid chemistry threatens to outpace governance infrastructure designed to ensure biosecurity and biocontainment. From simple base modifications in mRNA vaccines to fully synthetic genetic polymers invisible to natural biological systems, there is a continuum of synthetic nucleic acid chemistries, each presenting distinct considerations for risk governance and policy. This continuum can be conceptualised as a five-stage framework (Figure 1) characterising the progression from natural to fully orthogonal genetic systems. Stage 1 includes nucleoside substitutions like N1-methylpseudouridine (m1Ψ) in COVID-19 mRNA vaccines, which improved translational efficiency and reduced innate immune recognition (Nance and Meier, 2021; Morais et al., 2021). Stage 2 involves ribose sugar modifications like 2′-o-methyl, 2′-fluoro, 2′-O-methoxyethyl (MOE), and locked nucleic acid (LNA) chemistries that dominate currently approved antisense therapeutics (Crooke et al., 2018). These modifications confer enhanced nuclease resistance and improved target binding while maintaining compatibility with natural enzymatic machinery. Stage 3 involves the replacement of the phosphodiester linkages, which bridge consecutive nucleosides. Phosphorothioate linkages are commonly used in most approved oligonucleotide therapeutics (Crooke et al., 2018), where oxygen is substituted with sulphur to extend oligonucleotide half-life from minutes to days. For example, nusinersen for spinal muscular atrophy (Finkel et al., 2017) and inotersen for hereditary transthyretin amyloidosis (Benson et al., 2018; Crooke et al., 2018) are both approved antisense oligonucleotide therapeutics. Phosphorodiamidate morpholino oligomers (PMOs) are a relatively new class of backbone modifications that substitute morpholine rings for ribose sugars and have already been incorporated in four FDA-approved exon-skipping treatments for Duchenne muscular dystrophy (Egli and Manoharan, 2023). Peptide nucleic acids (PNAs) replace entire sugar-phosphate backbone with pseudopeptide linkages (Saarbach et al., 2019).

Stages 4 and 5 represent a more fundamental departure from natural nucleic acid architecture. These approaches are largely incompatible with endogenous replication and degradation processes while retaining the capacity to bind therapeutic targets such as proteins and receptors. However, they require further development before they become technically feasible as therapeutics. Stage 4 is made of xenonucleic acids (XNAs) that are synthetic genetic polymers where the sugar-phosphate backbone is replaced with non-natural chemical structures. Recent scientific advances in the field have demonstrated up to eight distinct XNA chemistries capable of storing genetic information and supporting heredity (Chaput, 2019; Chaput, 2021). Concomitantly, researchers showed that some XNAs can undergo Darwinian evolution (Pinheiro et al., 2012) and act as enzyme catalysts (Taylor et al., 2015). However, since natural polymerases cannot recognise or process XNA backbones, replication and amplification of XNAs require specially engineered polymerase enzymes along with synthetic building blocks. Stage 5 comprises mirror-image (L-configured) oligonucleotides which share the same molecular composition as natural oligonucleotides but are arranged in the opposite 3D orientation. This structural inversion makes these molecules invisible to the enzymes that normally recognise, copy, and degrade genetic material (Young et al., 2019). Researchers have harnessed this biological invisibility for effective long-lasting therapeutic applications, while preserving their capacity to bind therapeutic targets such as proteins. For example, Spiegelmers are mirror-image aptamers, engineered to bind specific disease targets/proteins, that persist in the bloodstream for days rather than minutes (Vater and Klussmann, 2015). More recently, researchers have demonstrated individual proof-of-concept steps toward mirror-image replication, including the synthesis of mirror-image enzymes capable of copying short mirror-image DNA sequences in controlled laboratory conditions (Xu and Zhu, 2022; Adamala et al., 2024). While significant progress has been made, a complete mirror-image organism remains a distant prospect requiring solutions to hundreds of unresolved problems across chemistry, biology, and engineering. Stage 4 and 5 molecules represent genuinely transformative scientific advances but require extensive research and development to be produced at scale.

### 3.2 Enabling infrastructure–synthesis and detection capabilities

The governance questions raised by the orthogonality spectrum described in Section 2.1 are not merely theoretical: they are urgent because advances in synthetic nucleic acid technology have progressively opened access to increasingly complex chemistries. The ability to synthesise custom DNA sequences has transformed from a specialised laboratory skill into a commodity service, and likewise, this may happen for modified nucleic acid architectures. Current gene synthesis costs have fallen from approximately $25 per base pair in 1999 to under $0.15 today (Hughes and Ellington, 2017; Carlson, 2025). Benchtop devices can now assemble constructs up to several kilobases without hazardous reagents or specialist training. The Nuclear Threat Initiative projected in 2023 that desktop synthesisers would give users (with limited laboratory skills) access to constructs up to 7,000 base pairs long (Carter et al., 2023). For Stage 1 chemistries, this accessibility is largely a feature where established commercial suppliers routinely incorporate these modifications into synthesised sequences at scale. In comparison, Stage 2 and 3 modifications are considerably more demanding at scale, where 2′-fluoro, phosphorothioates, LNA, or PMOs, require specialised phosphoramidite chemistry, carefully optimised coupling conditions, and quality control methods that go well beyond standard oligonucleotide synthesis (Egli and Manoharan, 2023). In practice, this means that while Stage 2 and 3 therapeutics are manufactured commercially, doing so reliably requires significant technical expertise and dedicated equipment. These chemistries are not yet accessible to the broad user base that now routinely orders natural DNA sequences.

The novel chemistries of Stage 4 and 5 molecules warrant a different analytical framework entirely, as their departure from natural nucleic acid architecture places them in a distinct category from the modifications discussed above. In Stage 4, this involves synthetic chemical divergence from the natural population, and in Stage 5, it involves reversed stereochemistry. Both XNA and mirror molecules require building blocks that are not available commercially at scale. Even the polymerases needed to copy XNA templates must be individually engineered for each chemistry, and synthesis yields remain low enough to restrict serious work to a small number of highly specialised laboratories worldwide (Maola et al., 2024; Christensen et al., 2022). Similarly, mirror-image oligonucleotides can be chemically synthesised in small quantities, but scaling beyond research quantities is technically and economically prohibitive at present (Vater and Klussmann, 2015). For now, the inaccessibility of these chemistries functions as a practical containment mechanism.

Current biosecurity screening coordinated via the International Gene Synthesis Consortium (IGSC) has been designed around the synthesis landscape as it exists for Stages 1–3. The Harmonised Screening Protocol introduced by IGSC requires orders above 200 base pairs to be checked against regulated pathogen sequence databases, where sequence identity is the primary indicator of risk (Carter et al., 2014). Even within this natural sequence framework, coverage is incomplete: roughly 20% of global synthesis capacity sits outside the IGSC, no country currently mandates screening as a legal requirement. A recent study found that only one of 38 providers flagged an order containing fragments of the 1918 influenza virus (Edison et al., 2026). While not of immediate concern, developing a biosecurity landscape that accounts for non-natural Stage 4 and 5 chemistries seems prudent. The groundwork for such frameworks can be laid now, while these chemistries remain largely confined to specialised laboratories.

### 3.3 XNA-based therapeutics–reality vs. technical feasibility

Stage 4 and 5 based therapeutics are an emerging and promising class of biocontained molecules. The most clinically advanced examples are Spiegelmers (Stage 5 mirror-image aptamers) developed by NOXXON Pharma. Currently, three Spiegelmer candidates have completed Phase 2a trials: NOX-A12 in chronic lymphocytic leukaemia and glioblastoma, NOX-E36 in diabetic kidney disease, and NOX-H94 in anaemia of chronic inflammation (Ludwig et al., 2017; Menne et al., 2017). Their mirror-image chemistry confers plasma half-lives in the range of 38–53 h and eliminates the immune reactions that complicate many natural nucleic acid therapies (Vater and Klussmann, 2015). XNA-based aptamers have also demonstrated strong binding against targets including the SARS-CoV-2 spike protein (Lozoya-Colinas et al., 2023). However, rapid development of these therapeutics remains constrained by stage-specific technical barriers. For XNA systems, the primary roadblocks are the requirement for specially engineered polymerases that are far slower than their natural counterparts and the absence of building blocks at commercial scale (Maola et al., 2024; Christensen et al., 2022). For Spiegelmers, chemical synthesis of mirror-image oligonucleotides remains technically demanding and economically prohibitive beyond research quantities (Vater and Klussmann, 2015).

These same barriers constitute an inadvertent biosecurity layer. Since natural enzymes cannot replicate or process XNA, genetic material from XNA-based systems is unlikely to be functionally integrated into recipient cells during genetic exchange events. This reduces the likelihood of engineered sequences entering ecological circulation, illustrating how technical immaturity acts as *de facto* biocontainment (Gómez-Tatay and Hernández-Andreu, 2024). A natural question follows: will the pace of scientific advance in Stage 4 and 5 technologies outstrip the development of adequate governance frameworks? The trajectory of earlier stages offers a useful precedent. As the rapid democratisation of natural DNA synthesis has shown us, technically prohibitive technologies today tend to become routine within a decade. Therefore, the current limitations of Stage 4 and 5 chemistries offer a meaningful opportunity to develop governance frameworks ahead of need.

### 3.4 From mRNA stability to mirror life–risk escalation and oversight

Each step beyond natural nucleic acid chemistry adds to a biosecurity-relevant feature combining enhanced environmental persistence, reduced immune recognition, and increasing invisibility to existing surveillance tools. Stage 3 modifications already extend oligonucleotide persistence in tissues from hours to months (Crooke et al., 2018), while Stage 4 XNA systems are nuclease-resistant, invisible to standard sequencing, and unable to be amplified by any conventional method (Taylor et al., 2015). Mirror life represents the theoretical endpoint of this trajectory: organisms built entirely from mirror-image biomolecules. Consistent with the distinction drawn above, this is an increase in orthogonality rather than a uniform increase in danger. There may be interesting trade-offs in these risks; the earlier stages are more likely to be recognised by immune systems, so may trigger strong immune responses. Later stages are still mostly hypothetical, but it is plausible that they will not trigger as strong immune responses until they cause mechanical damage or spread ecologically due to their lack of compatibility with potential vectors. The effects and risks of creating these entities are unclear, which has grounded the response to their possibility.

The scientific groundwork for creating Stage 5 architectures remains at an early stage, with researchers demonstrating individual proof-of-concept steps such as the chemical synthesis of a mirror-image RNA polymerase capable of transcribing full-length mirror-image ribosomal RNAs in controlled laboratory conditions (Xu and Zhu, 2022). The scientific community has already responded proactively, with a 2024 call in *Science*—before the capability is realised—for a moratorium on research that would result in a mirror bacterium or other self-replicating organism (Adamala et al., 2024). The UNESCO International Bioethics Committee endorsed this position in July 2025 (James and Cai, 2025). Importantly, Adamala et al. did not propose restricting research on the further development of individual mirror molecules or the ability to create them, only their being combined into a self-replicating system. The governance implications of this continuum merit particular attention. Progression through successive stages increasingly challenges existing biosecurity frameworks largely designed around natural genetic systems. Currently approved therapeutics span Stages 1–3, with backbone-modified drugs such as nusinersen (Finkel et al., 2017) and inotersen (Benson et al., 2018) already well established in clinical practice. As XNA/mirror polymerase engineering continues to advance, new therapeutic candidates spanning Stages 4 and 5 may emerge within the next decade. The modifications that extend therapeutic half-lives also extend environmental persistence, and the orthogonality that enables biocontainment also makes surveillance difficult. The plasma half-lives of Spiegelmers (38–53 h), though dramatically extended relative to natural aptamers, may reflect renal excretion of intact molecules rather than degradation (Boisgard et al., 2005), a distinction with meaningful environmental implications. There is standing evidence that modified oligonucleotides are excreted substantially intact in urine (Jo et al., 2023). Unlike degraded fragments, intact mirror-image oligonucleotides excreted into wastewater would be nuclease-resistant and undetectable by standard sequencing. The environmental persistence of excreted Spiegelmers has not been directly studied, which represents a research gap. Therefore, establishing environmental half-life baselines, first for currently approved modified oligonucleotides and then for mirror-image and XNA chemistries, should be a near-term research priority. The necessary assays are tractable by analogy with existing work on the environmental durability of dsRNA biopesticides, which had to establish persistence baselines for a novel nucleic-acid class in soils and waterways (Luo et al., 2024).

Position on the spectrum is a rough proxy for risk rather than a direct measure of it. Instead, risk depends on three underlying properties that a molecule may acquire as it moves along the spectrum. The first is resistance to degradation and clearance (from tissue or environment), which determines how long a molecule persists. The second is invisibility to recognition and surveillance, which covers both biological detection by the immune system and technical detection by sequence-based screening. This invisibility is driven by chirality and backbone novelty rather than by stability, which is why a mirror molecule can escape detection for reasons unrelated to how long it persists. The third is autonomous replication, which is categorical rather than graded, unlike the first two properties. The concern is not whether a molecule can be copied, since engineered polymerases can already copy XNA templates in the lab. The concern is whether orthogonal components form a system that replicates itself without outside enzymes or substrates. This self-sustaining autonomy is approached only at Stage 5 and marks the categorical threshold. In this regard, the core consideration for governance is not evasion on its own but the durability of that evasion, which becomes relevant only when a molecule can escape recognition and persist. Evasion by itself is not the concern, since a Stage 1 modification such as m1Ψ already dampens immune recognition, and that is a therapeutic goal rather than a hazard. Table 1 maps these properties onto the five stages, demonstrating where crossing a threshold on one of them should prompt closer monitoring and staged review. Crossing a threshold in this sense signifies that the molecule warrants more governance attention, not a judgement that it has become dangerous. We suggest here that persistence warrants additional oversight once tissue or environmental half-life extends significantly beyond natural degradation timescales, and invisibility warrants oversight when a molecule escapes the sequence-based bio-surveillance screens.

**TABLE 1:** Governance-relevant properties mapped onto the five-stage spectrum. For each stage, the table gives the dominant chemical change and the level of three properties that inform risk assessment and oversight: resistance to degradation and clearance (persistence), invisibility to recognition and surveillance, and autonomous replication. Persistence and invisibility are graded properties that generally increase across the spectrum. Autonomous replication is categorical rather than graded, depending on whether a self-replicating system is probable or not.

Stage	Dominant chemical change	Persistence	Invisibility (escapes detection)	Autonomous replication
1	Nucleobase modification	Low	Low (evades innate recognition but evasion is short-lived)	None
2	Sugar modification	Moderate	Moderate	None
3	Backbone modification	High (tissue persistence)	Moderate	None
4	XNA	High	High (invisible to standard sequencing)	Engineered, *in vitro*; needs synthetic polymerase and substrates
5	Chirality	High	Maximal (invisible to natural enzymes and to sequencing)	Potentially autonomous if assembled into a replicating system

Overall current frameworks, built around sequence homology screening and standard sequencing, are increasingly misaligned with the emerging chemistry they are meant to govern. These methods focus on sequence identity rather than the chemical architecture (i.e., backbone, nucleobase, or chirality modifications) that defines stage 3–5 technologies. This is reflected by the fact that later stages will escape detection by standard sequencing methods, requiring different detection protocols. Some screening measures are available within current regulatory structures, such as chemistry-aware flags in the International Gene Synthesis Consortium’s Harmonised Screening Protocol. However, the precise thresholds for the graded properties of persistence and invisibility depend on pharmacokinetic and environmental baselines that do not yet exist but should be the focus of near-term research priority. In fact, the 2024 *Science* consensus on mirror life research explicitly recommends developing detection methods and biosurveillance systems to enable governance and biosafety that anticipate technological capabilities (Adamala et al., 2024). This principle applies equally across the modification spectrum: governance frameworks should engage proactively with the trade-offs embedded in it, rather than waiting for capabilities to emerge and expand that current infrastructure cannot detect.

## 4 Discussion

### 4.1 Engineering biology involves trade-offs

Optimisation is often treated as an unqualified positive feature of design. Novel synthetic designs aim for stability and orthogonality, with good reason; they allow us to design organic systems more effectively. If a molecular structure is orthogonal, it will do exactly what we want without further causal effects, allowing us to build systems with precise causal sequences from orthogonal components (Woolfson, 2026). Optimising such structures to directly and stably do our intended purpose makes sense, but it is worth recognising that tasks, including mRNA stabilisation, are not optimisation tasks with a single global maximum. There are risk–benefit trade-offs across biology and public communication. This is because biological design is deeply unorthogonal, creating a mismatch between our design aims and biology as it exists.

Biological systems are built through contingent, path-dependent sequences, which often make them less modular and more integrated in unexpected ways. Biological causation is often structured more like a cascade, with feedback systems being triggered that modulate, amplify, or/and dampen the effects (Ross, 2026). These feedback systems are crucial for the regulation of responses, the degradation of upregulating causal factors, and immunological responses to novelty. Biological structures do not have the hallmarks of human design, like sequences or structures optimised by conscious testing and iteration. Instead, organisms’ features are life-history specific, the product of a developmental trajectory of which each stage influences the next, and each stage has unique environmental signatures. These sequences are built on each other, making many biological features deeply canalised and unable to be easily altered or removed. As natural selection cannot occur with foresight, sub-optimal forms cannot be maintained for the later production of a more optimal outcome. The deeply embedded nature of biological composition and the lack of foresight in design create many of the wild and beautiful designs we see.

Designing biological systems can either fight these constraints or work with them. Engineering is often considered a process that starts with a design posited through first principles and works systematically to meet that design, creating something repeatable and scalable. In synthetic biology, this involves replacing the contingent, irrational design of natural selection with rationally designed, predictable systems (Endy, 2005). Orthogonality is a necessary component of this type of design. Kludging, in contrast, prioritises current function over systematic structure (O’Malley, 2009; O’Malley, 2011). It accepts that biology involves interdependencies and complexities, and rather than fighting against biology, it works with its constraints. It involves iterative design, tweaking, retrofitting, and debugging. Given this process, solutions in one domain may not translate to another. Kludging accepts biological design as it is, working with its constraints rather than aiming to build something completely novel and streamlined. Both design methodologies are important, but orthogonality and stability, when taken to the extreme, can have predictable negative implications.

Mirror life and mRNA stabilisation involve redesigning nucleic architecture, but mRNA stabilisation is closer to kludging, whereas mirror life involves drastic redesign. Stabilisation involves the iterative tinkering of nucleic acid design. Modified nucleosides, altered cap structures, codon optimisation, and delivery refinements adjust how molecules behave inside already evolved cellular machinery rather than escape these relationships. In contrast, mirror life’s redesign of nucleic architecture is aimed at creating a separate design from standard biological pathways.

Mirror life’s autonomy was viewed as an advantage, as it would provide a toolbox for creating novel designs, since xenobiological designs could not interact with extant biological systems (Schmidt, 2010). It is, however, now recognised that it poses unique biotic risks. These risks appear to have been extended only so far as the point at which a self-replicating orthogonal system can arise (Schmidt, 2010; Gómez-Tatay and Hernández-Andreu, 2024; Adamala et al., 2024). This stance does not fully recognise that natural systems are evolved systems and their entrenched interactions can be disrupted by high orthogonality. We suggest that any extremely orthogonal system poses risks that warrant monitoring when interacting with natural biology.

Persistence and xenobiology designs span a continuum (see Section 2). This continuum is the degree to which the organic entities we create are orthogonal to existing metabolic pathways, enzymatic degradation systems, and immune systems. Experimental XNA systems (Pinheiro et al., 2012) and semi-synthetic organisms with expanded alphabets (Malyshev et al., 2014) both display partial orthogonality. The partially orthogonal systems required for therapeutics must thread the needle by being orthogonal to parts of cellular machinery, to avoid detection and degradation, while remaining non-orthogonal to other parts, such as the ribosomes. If this partial orthogonality is inaccurate, it could trigger novel immune responses through unfamiliar molecular patterns or disrupt homeostatic relationships through prolonged expression that natural regulatory systems were not calibrated to manage. If a design escapes all the cascading feedback systems that regulate and degrade biological factors, it also escapes all of life’s evolved means of controlling and integrating novelty. This is at the core of the trade-off in biotic design; perfect optimisation of one biotic function will often yield dysfunction across other modalities and make a less robust system (Wimsatt, 2007). Inefficiency in the persistence of designed mRNA could be viewed as a desirable part of its design. Inefficiency means controlled degradation and partial immune recognition can occur, which is evolutionary continuity rather than a design failure.

The kludging/engineering distinction carries a direct governance implication. Because the stabilisation of delivery systems proceeds by iterative modification of existing biology rather than discontinuous redesign, any risks it generates will not arrive as a single identifiable threshold event. This is different from the mirror life case, where a discrete capability (a self-replicating mirror organism) can serve as a clear regulatory target. Incremental stabilisation is currently governed under biosafety frameworks built for commercial biotechnology. In contrast, following the recommendations of UNESCO and the National Academies, orthogonal xenobiology requires stricter biosecurity frameworks (National Academies of Sciences, Engineering, and Medicine, 2018; James and Cai, 2025).

There should be efforts to bridge this monitoring gap, because if iterative novel therapeutic designs increase persistence by completely insulating the delivery system from the host regulatory system, then they would approach xenobiological design. Conceptual and empirical work should be committed to determining where wise limits should be placed between more standard stabilisation strategies and more biosecurity-risky designs. Anticipatory governance, therefore, requires not just rules about which architectures are permissible, but staged oversight mechanisms tied to specific orthogonality-relevant parameters. The relevant parameters are those that track progress along the trajectory mapped in Section 2: half-life extension, nuclease resistance, immune evasion efficacy, and environmental persistence. Regulatory review should be triggered by measurable advances across these parameters, rather than waiting for the appearance of a novel endpoint that existing frameworks were not designed to recognise.

## 5 Risk management

### 5.1 Containment of novel structures

Novel nucleic acid architectures could have a range of environmental effects, functioning as irritants or antigens, or possibly being incorporated into other species. There are a range of reasons for developing updated Biosafety protocols for creating novel nucleic acid architectures.

Existing regulatory frameworks for nucleic acid therapeutics are product-focused and sequence-focused. They assess individual therapeutic candidates as they move through defined development pipelines and screen for risk by comparing sequences against known pathogen databases. This architecture was designed for a landscape in which the primary biosecurity concern was the misuse of known biological agents. It was not designed to track cumulative progress along an orthogonality trajectory across multiple independent research programs and product classes, which will result in novel design (c.f. Kelle, 2013; Carter et al., 2014; Epstein et al., 2025). While no general measure of orthogonality currently exists, developing such metrics would be fruitful for building a research programme to categorise the relationship between natural and designed biology and to determine when escalated oversight is required. Orthogonality-based screening should be developed to complement the sequence homology screening currently used by IGSC. More broadly, the development of detection and surveillance methods for novel nucleic acid chemistries should parallel the development of those chemistries themselves. The same research programs that advance synthetic capabilities should be expected to contribute to the methods needed to detect and monitor their products.

Detection will be important as the volume of biomass produced through experimentation increases, creating opportunities for release. Standardly, for mRNA vaccine production, research is recommended to be conducted in a Biosafety Level 1 (BSL-1) lab. In some instances, research involves manipulating infectious agents that require BSL-2 or BSL-3 containment. This involves the sterilisation of all recombinant and synthetic materials, usually by autoclaving or chemical decontamination (often bleach). This process is often quite effective at destroying recombinant DNA, as breaking the DNA into progressively smaller chemical fragments makes it highly unlikely that any coding sequences will escape into nature. We do, however, know that this can happen.

Standard biosafety decontamination is designed to kill organisms, not to completely destroy nucleic acid sequences. Research on laboratory DNA waste has demonstrated that standard methods such as autoclaving leave amplifiable sequences intact, and that decontamination procedures effective against microorganisms are different from those required for nucleic acid elimination (Gefrides et al., 2010). Moon (2025) extends this concern to genetically engineered microbes more broadly, noting that biocontainment frameworks have yet to adequately address the environmental persistence of released genetic material following standard disposal. For novel nucleic acid architectures, this problem is compounded as existing environmental surveillance infrastructure, built around sequence homology screening and standard sequencing methods, will be blind to them. Stage 3 backbone modifications already resist standard nuclease degradation, and Stage 4 and 5 architectures cannot be detected by conventional sequencing at all. Containment protocols and environmental monitoring will therefore need to be developed in parallel with the chemistries themselves, rather than retrofitted after deployment.

The biocontainment of novel nucleic architectures differs from that of recombinant DNA. RNA is more subject to degradation than recombinant DNA, as its nucleic backbone is more subject to cleavage, meaning any surviving RNA fragments would likely be highly degraded, non-replicative, and non-functional. For mRNA therapeutics specifically, the relevant containment question is not whether stabilised sequences survive autoclaving. Current stabilisation strategies extend intracellular half-life rather than resistance to heat or oxidative degradation, making post-sterilisation survival unlikely. More relevant is persistence within living systems: stabilised mRNA that may accumulate in tissues and be excreted intact, creating opportunities for entry into the environment. This represents a release pathway that standard biosafety protocols were not designed to address. Such issues are unlikely to be pressing under current designs but are important for anticipatory governance as we push into further novel designs and increasing volumes of these designs.

### 5.2 Human trials

First-in-human trials of novel therapies pose significant risks and uncertainties to volunteers. Infamous cases include, the death of Jesse Gelsinger in 1999 after a trial of gene therapy for his (relatively mild) genetic disorder (Savulescu, 2001), the “elephant man” trial of novel immunotherapy in 2006 resulting in intensive care and loss of fingers and toes for several volunteers (Hopkin, 2006), and a trial of a neurological medication in 2016 that resulted in coma among several volunteers and one death (Chaikin, 2017). Several lessons have been drawn from these outcomes (Chaikin, 2017). First, appropriate toxicology studies should be conducted in animals, and extreme care should be taken to assess risks to early human volunteers based on animal data. Second, initial trials of experimental therapies should enrol participants with severe disease as compared to those with milder forms of relevant diseases who have a more favourable prognosis and thus more to lose from irreversible harms due to experimental treatments. Third, initial volunteers should be exposed to experimental agents slowly and monitored for toxicity before exposure of more volunteers or escalation of dosage, rather than multiple volunteers being exposed at the same time. Fourth, experimental agents should be progressed through clinical trials in a manner that prevents premature advancement to Phase 1 in the case of toxicity (Taylor, 2025).

The COVID-19 vaccine data also raises a question directly relevant to novel stabilised architectures: whether repeat exposure to modified mRNA structures can sensitise the immune system over time. Adverse reactions, including myocarditis and pericarditis, appeared more frequently following the second dose, suggesting prior exposure had altered the immunological response (Rosenblum et al., 2022). These effects were disproportionately experienced by the young, raising questions around the ethics of mandates (Funk et al., 2022; Bardosh et al., 2024). The mechanism for these effects remains debated; the LNP and antigen coding region appear to be the more likely mediators (Xie et al., 2023; Zelkoski et al., 2025). But the pattern raises a question that the COVID-19 data cannot resolve: whether cumulative exposure to multiple distinct mRNA therapeutics across a patient’s lifetime could produce analogous sensitisation through different mechanisms. This is particularly relevant for stabilised architectures intended for chronic or repeated administration, where extended persistence may prolong immunological interaction beyond anything current vaccine data captures.

First-in-human trials of mRNA therapeutics should be informed by these findings, as well as the toxicities observed from mRNA interventions to date. First, toxicology studies should be conducted in appropriate animal models, with the duration of these studies to be extended in proportion to the stabilisation of mRNA and the likely duration of effects. Second, early trials of therapeutics, as opposed to vaccines, should enrol patients with the illness in question, and a risk-benefit assessment should inform prioritisation of patients who are most likely to face a favourable benefit-to-risk ratio. Third, initial trials should involve exposure of one or a small number of volunteers at a time, with an appropriate duration of monitoring for toxicity prior to progressive exposure of greater numbers of participants. Fourth, if or when stable mRNA therapeutics advance towards licensure, ongoing monitoring should trigger review of development pathways if any significant toxicities are identified, especially if these are similar to previously observed toxicity from mRNA or similar interventions.

Further, there should be prospective and ongoing assessment of the extent to which more stable forms of mRNA persist in humans and in the general environment, including through testing and eventual use in humans. If there is evidence of accumulation of stable mRNA in humans or other parts of an ecosystem, then further research should be conducted regarding any lasting effects this might produce.

### 5.3 Communicative risk

As mentioned in 1.3, communication about mRNA, particularly the stabilisation process, poses dual communication challenges when engaging with the public:

1. Avoiding narratives of permanence and determinism, and
2. Maintaining the public trust that enabled the success of mRNA vaccines.

To address (1), media communication about mRNA requires clarity about the relationships and distinctions between mRNA and DNA–to avoid genetic essentialist biases being imported into interpretations of mRNA applications. Lessons can be taken from the literature on science communication about genetics, which suggests the avoidance of deterministic language and metaphors like ‘destiny’ ‘fate’ and ‘blueprint’ (Nelkin and Lindee, 1995; Nelkin, 2001). In addition to modifying media communications, education is likely to play a role in successful communication of mRNA applications. Here, we can again learn from the genetics literature. Some have suggested that early school education shapes genetic essentialist beliefs, and that interventions on genetic education, including a greater emphasis on the interactions between genes and environment, can ameliorate these underlying beliefs and attitudes (Jamieson and Radick, 2017). There is also evidence that genetic knowledge and genetic education can mitigate genetic essentialism and that genetic knowledge, as well as general education, correlates with positive attitudes and perceived acceptance of gene therapies (Aiyegbusi et al., 2020; Dar-Nimrod et al., 2021). This suggests an additional avenue to prevent public misconceptions about mRNA technologies: Increased education about mRNA and its relationship to DNA.

We thus make the following recommendations for communicating about mRNA therapeutics and technologies:

1. Lexical choices that avoid essentialist and deterministic connotations. This includes deterministic and fatalist language, such as “fate”, “destiny”, or “inevitable” or “immutable”
2. Clear communication about the stability of mRNA, ensuring that communication about longer lasting and more stable mRNA components are not misunderstood as being permanent and immutable, especially with regard to permanent and immutable downstream effects on the body.
3. Clear communication about the relationships with and distinctiveness of DNA and mRNA to avoid conflating the two and invoking genetic essentialist beliefs about mRNA altering one’s identity.

## 6 Conclusion

mRNA stabilisation represents one of the most promising frontiers in programmable biology. Designed orthogonality has increased mRNA’s resilience and translation efficiency, making it an effective vehicle for vaccines and, in the future, novel therapeutics and industrial applications. This effectiveness and potential still warrants an obligation to monitor how incremental increases in orthogonality will affect us both biologically and in terms of public perception of biotechnology.

The analogy with mirror life clarifies the use and risk of orthogonality in biology. If there are degrees of orthogonality and we can incrementally increase the number of technologies that utilise orthogonality or increase the orthogonality in technologies, we will likely increase risk. By focusing on this conceptual link, we hope to improve governance. We can create governance for techniques to increase biological stability, immunoevasion, and persistence, but if we do not recognise that these are all united as forms of orthogonality, there will be governance gaps. These gaps will occur because restricting a new technique to increase orthogonality will likely leave an opportunity for a new technique to be developed that avoids the governance mechanisms. Thus, recognising the unified nature of this problem provides productive means for addressing the risks we will face.

We are not implying that mRNA stabilisation is dangerous in the way mirror life would be; rather, that cumulative intervention to create increasingly orthogonal and novel biotic architectures warrants foresight and governance. Increasing orthogonality will have many therapeutic uses, beyond just stabilising mRNA. We will want to explore these novel effects, but we must pay attention to the risk–reward trade-offs at every step. The capacity to design molecules that evade biological recognition is becoming more accessible. Governance will need to be pre-emptively prepared for a stepwise increase in orthogonality, beyond mRNA to the broader class of synthetic nucleic acid architectures that existing biosecurity infrastructure was not built to govern (also see Epstein et al., 2025). Equally, pre-emptive risk mitigation strategies should be employed that consider the ethics of human trials, and the public response to mRNA technologies and their implementation. Lessons can be learned from less successful risk mitigation in these areas, such as public responses to COVID-19 vaccine roll-outs, and the enduring legacy of genetic essentialism from science communication and education of genetics.

This pre-emptive governance requires a shift from product-focused, ‘patchwork’ approaches to trajectory-focused oversight (Kelle, 2013). Current frameworks assess individual therapeutic candidates against known risk profiles. We recommend that research programs and product classes should trigger regulatory review when they progress along orthogonality-relevant parameters. We have suggested possible parameters, including half-life extension, nuclease resistance, and immune evasion efficacy. This applies most immediately to mRNA, simply because it is the subject of the most active research, but the same should apply to other research platforms. Anticipatory governance for biotechnology requires that biosecurity and bioethics communities engage with the orthogonality trajectory, in its entirety, rather than respond reactively to each new chemistry as it emerges.

## Data Availability

The original contributions presented in the study are included in the article/supplementary material, further inquiries can be directed to the corresponding author.
